# Identification of 5 novel germline *APC *mutations and characterization of clinical phenotypes in Japanese patients with classical and attenuated familial adenomatous polyposis

**DOI:** 10.1186/1756-0500-3-305

**Published:** 2010-11-16

**Authors:** Hong Tao, Kazuya Shinmura, Hidetaka Yamada, Masato Maekawa, Satoshi Osawa, Yasuhiro Takayanagi, Kazuya Okamoto, Tomohiro Terai, Hiroki Mori, Toshio Nakamura, Haruhiko Sugimura

**Affiliations:** 1 First Department of Pathology, Hamamatsu University School of Medicine, 1-20-1 Handayama, Higashi Ward, Hamamatsu, Shizuoka 431-3192, Japan; 2 Department of Laboratory Medicine, Hamamatsu University School of Medicine, 1-20-1 Handayama, Higashi Ward, Hamamatsu, Shizuoka 431-3192, Japan; 3 First Department of Medicine, Hamamatsu University School of Medicine, 1-20-1 Handayama, Higashi Ward, Hamamatsu, Shizuoka 431-3192, Japan; 4 Department of Surgery, Fujinomiya City General Hospital, 3-1 Nishiki-cho, Fujinomiya, Shizuoka 418-0076, Japan; 5 Second Department of Surgery, Hamamatsu University School of Medicine, 1-20-1 Handayama, Higashi Ward, Hamamatsu, Shizuoka 431-3192, Japan

## Abstract

**Background:**

Familial adenomatous polyposis (FAP) is an autosomal dominant hereditary disease characterized by multiple colorectal adenomatous polyps and frequent extracolonic manifestations. An attenuated form of FAP (AFAP) is diagnosed based on a milder colorectal phenotype, and the colorectal phenotype of (A)FAP has been linked to germline *APC *mutations. The relationships between the spectrum of mutations and extracolonic manifestations are quite well known, but they need to be further defined.

**Findings:**

Nine germline *APC *mutations, but no large deletions, were identified in the *APC *locus of 8 (A)FAP patients, and 5 of the mutations, c.446A > T (p.Asp149Val), c.448A > T (p.Lys150X), c.454_457insAGAA (p.Glu152ArgfsX17), c.497insA (p.Thr166AsnfsX2), and c.1958G > C (p.Arg653Ser), were novel mutations. In one patient the p.Asp149Val mutation and p.Lys150X mutation were detected in the same *APC *allele. The c.1958G > C mutation was located in the last nucleotide of exon 14, and RT-PCR analysis revealed that the mutation resulted in abnormal splicing. The above findings meant that a nonsense mutation, a frameshift mutation, or an exonic mutation leading to abnormal splicing was found in every patient. The following phenotypes, especially extracolonic manifestations, were observed in our (A)FAP patients: (1) multiple gastroduodenal adenomas and early-onset gastric carcinoma in AFAP patients with an exon 4 mutation; (2) a desmoid tumor in two FAP patients with a germline *APC *mutation outside the region between codons 1403 and 1578, which was previously reported to be associated with the development of desmoid tumors in FAP patients; (3) multiple myeloma in an AFAP patient with an exon 4 mutation.

**Conclusions:**

Nine germline *APC *mutations, 5 of them were novel, were identified in 8 Japanese (A)FAP patients, and some associations between germline *APC *mutations and extracolonic manifestations were demonstrated. These findings should contribute to establishing relationships between germline *APC *mutations and the extracolonic manifestations of (A)FAP patients in the future.

## Background

Familial adenomatous polyposis (FAP) is an autosomal dominant familial cancer syndrome characterized by the early onset of large numbers of adenomatous polyps throughout the entire colon and a nearly 100% lifetime risk of colorectal cancer (CRC) if the colon is not removed [1]. A small proportion of familial colorectal polyposis cases were recently found to be associated with biallelic germline mutations of the *MutYH *gene [2]. However most FAP cases are caused by germline mutations of the tumor suppressor gene *adenomatous polyposis coli *(*APC*), which encodes a 2843-amino-acid protein that contains a variety of functional domains involved in cell cycle control, differentiation, transcription, migration, and apoptosis [3]. More than 1000 pathogenetic mutations have been detected throughout the *APC *gene, and the lifetime penetrance of the disease is close to 100% [3-5]. Some FAP cases have been classified as 'attenuated FAP (AFAP)' because of their attenuated phenotypes. Although there is still no consensus as to the precise definition of AFAP, some papers have summarized the characteristics of AFAP as follows: development of far fewer colorectal adenomatous polyps in AFAP patients than in classical FAP and the onset of adenomatous polyps and colorectal cancer 10~15 years later in AFAP patients than in classical FAP [6-8]. The germline *APC *mutations in AFAP patients have been found to occur at the 5' end and 3' end and in a specific region of exon 9 of the *APC *gene, in contrast to the germline *APC *mutations in classical FAP patients, which are found in other locations [3,8-10]. Thus, the analysis of the sites and spectrum of germline *APC *mutations in patients with multiple colorectal polyps is very important to the proper management of (A)FAP.

A number of extracolonic phenotypic manifestations are associated with FAP: upper gastrointestinal tract polyps and cancer, desmoid tumors, thyroid cancer, hepatoblastoma, congenital hypertrophy of the retinal pigment epithelium (CHRPE), and other extracolonic malignancies [1,3]. One of them, CHRPE, only occurs in patients with germline *APC *mutations between codons 457 and 1444, and desmoid tumors develop only in patients having mutations between codons 1403 and 1578 [1,3]. Although the correlations between the germline *APC *genotypes and FAP phenotypes are well known, they need to be further defined. In this study we investigated the genes of 8 Japanese (A)FAP patients for germline *APC *mutations, and we identified 9 germline *APC *mutations, including 5 novel ones. We also discuss possible relationships between the germline *APC *mutations and extracolonic manifestations in our (A)FAP patients.

## Methods

### Subjects

Blood samples were obtained from 5 patients with classical FAP and 3 patients with attenuated FAP, all of whom appeared to be unrelated, in the hospital of Hamamatsu University School of Medicine. Written informed consent was obtained from every patient. Lymphocyte genomic DNAs were extracted from the blood samples with a QIAamp DNA Blood Maxi kit (QIAGEN, Hilden, Germany). This study was approved by the Institutional Review Board of the Hamamatsu University School of Medicine (18-4).

### Conventional sequencing analysis of the *APC *gene

The 1st-15th exons of *APC *and their boundary regions were amplified by polymerase chain reaction (PCR) and directly sequenced with ABI BigDye Terminator Ready Reaction Mix (Applied Biosystems, Foster City, CA, USA) and an ABI 3100 Genetic Analyzer (Applied Biosystems) [11]. Information regarding the PCR primers is available upon request. Subcloning of the PCR fragments was performed by using a pGEM-T Easy TA Cloning Kit (Promega, Madison, WI) according to the supplier's protocol.

### Calculation of splicing efficiency and detection of the exonic splicing enhancer (ESE) sequence

The splicing efficiency of the wild-type allele and the mutant-type allele of the patient with c.1958G > C mutation was predicted by using the Berkeley Drosophila Genome Project (BDGP) splice prediction program [12]. The effect of the exonic mutation on putative ESE sites was predicted by the ESEfinder software program [13]. ESEfinder is a web-based resource that facilitates rapid analysis of exon sequences to identify binding motifs for serine/arginine-rich (SR) proteins.

### *APC *mRNA transcript analysis

RNAs were extracted from blood samples with a PAXgene Blood RNA Kit (QIAGEN) and converted to first-strand cDNAs by using a SuperScript First-Strand Synthesis System for reverse transcription (RT)-PCR (Invitrogen, Carlsbad, CA, USA) according to the supplier's protocol [14]. RT-PCR was performed with a set of primers, i.e., 5'-aaa gac gtt gcg aga agt tg-3' for the sequence of exon 13 and 5'-caa acc tcg ctt tga aga ag-3' for the sequence of exon 15, and the products were separated on 2% agarose gel and stained with ethidium bromide before being examined with an ultraviolet imaging system. The splicing rates were evaluated by comparing the intensities of the two main bands detected in each sample by using ImageJ software (National Institutes of Health, USA) as reported previously [15].

### Multiple ligation-dependent probe amplification (MLPA) analysis

A MLPA kit (P043 APC) was purchased from MRC-Holland (Amsterdam, The Netherlands), and reactions were carried out according to the manufacturer's instructions. Probe ratios below 0.7 and above 1.3 are regarded as indicative of a decrease and increase, respectively, of gene dosage.

## Results

### Identification of 5 novel germline *APC *mutations

**Figure 1:**
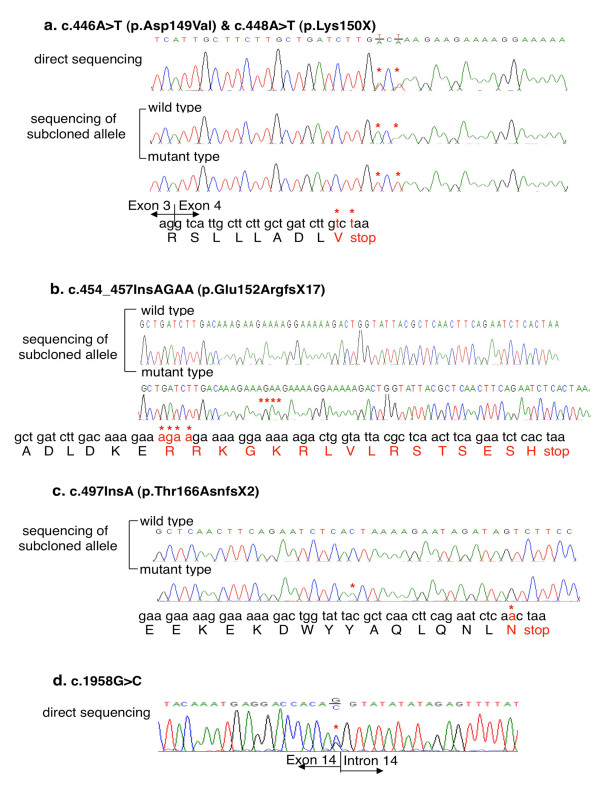
**5 novel germline *APC *mutations identified by sequencing analysis**. Results of sequencing the *APC *gene locus in DNA derived from the blood of Japanese (A)FAP patients. An asterisk indicates the location of the mutation. a. Results of sequencing analysis of the PCR product covering the c.446A > T and c.448A > T mutations. An amino acid sequence based on the mutated nucleotide sequence is shown below the electropherograms. b. Results of sequencing analysis of the subcloned PCR product covering the c.454_457insAGAA mutation. An amino acid sequence based on the mutated nucleotide sequence is shown below the electropherograms. c. Results of sequencing analysis of the subcloned PCR product covering the c.497insA mutation. An amino acid sequence based on the mutated nucleotide sequence is shown below the electropherograms. d. Results of sequencing analysis of the PCR product covering the c.1958G > C mutation. The boundary between exon 14 and intron 14 is indicated by a vertical line.

Genomic DNA sequencing of the entire *APC *coding regions and exon-intron boundaries enabled identification of a total of 9 germline *APC *mutations in 8 unrelated Japanese (A)FAP patients (Table 1). Five of the 9 germline *APC *mutations identified in this study, i.e., c.446A > T (p.Asp149Val), c.448A > T (p.Lys150X), c.454_457insAGAA (p.Glu152ArgfsX17), c.497insA (p.Thr166AsnfsX2), and c.1958G > C (p.Arg653Ser), had never been reported in any articles according to the information in the Human Gene Mutation Database or in the *APC *variant databases in the Leiden Open Variation Database (LOVD) [16-18], and none were found in a thorough review of the literature, indicating that they are novel mutations. Sequencing of the subcloned *APC *fragments revealed that the c.446A > T mutation and c.448A > T mutation detected in Patient 1 were located on the same *APC *allele (Figure 1a). Since the c.448A > T mutation is a nonsense mutation, the c.448A > T mutation is more likely than the c.446A > T missense mutation to be a disease-causing mutation. The c.454_457insAGAA mutation detected in Patient 2 and the c.497insA mutation detected in Patient 3 are frameshift type mutations and lead to the formation of premature stop codons (Figure 1b, c). The c.1958G > C mutation detected in Patient 4 was associated with an amino acid substitution (p.Arg653Ser) and was located in the last nucleotide of exon 14 (Figure 1d). The remaining 4 *APC *mutations had been reported previously [19-21].

**Table 1:** Germline *APC *mutations and clinical phenotypes identified in 8 Japanese (A)FAP patients

Patient ID	**Germline *APC *mutation**^**1 **^**(Exon**^**2**^**)**	Consequence	**Reference**^**3**^	**FAP type (age**^**4**^**)**	**Number**^**5 **^**of colorectal polyps**	Colorectal cancer (age)	**Extracolonic manifestation (age**^**4**^**)**
1	c.446A > T (4)	p.Asp149Val	This study	AFAP (34)	40-100	Absent (38^6^)	Gastric adenocarcinoma (34)
	c.448A > T (4)	p.Lys150X	This study				Multiple gastroduodenal adenomas(34)
2	c.454_457insAGAA (4)	p.Glu152ArgfsX17	This study	AFAP (29)	40-100	Present (51^6^)	Gastric hyperplastic polyps (29)
3	c.497insA (4)	p.Thr166AsnfsX2	This study	AFAP (69)	> 100	Present (69^4^)	Multiple gastroduodenal adenomas (69) Multiple myeloma (69)
4	c.1958G > C (14)	p.Arg653Ser	This study	FAP (32)	> 300	Present (32^4^)	Multiple gastroduodenal adenomas (32)
		Aberrant splicing					Duodenal adenocarcinoma (32)
5	c.1993_1994delTT (15)	p.Leu664IlefsX8	[19]	FAP (28)	> 300	NA^7^	Desmoid tumor (31) Multiple gastroduodenal adenomas (41) Small intestinal adenocarcinoma (41)
6	c.3505_3509delGAGAA (15)	p.Glu1169ThrfsX8	[20]	FAP (19)	> 300	Absent (19^6^)	Multiple gastroduodenal polyps (19)
7	c.3747C > A (15)	p.Cys1249X	[21]	FAP (22)	> 1000	Present (22^4^)	Multiple gastroduodenal adenomas (22) Desmoid tumor (25)
8	c.3927_3931delAAAGA (15)	p.Glu1309AspfsX4	[21]	FAP (31)	> 1000	Present (31^4^)	Multiple gastroduodenal polyps (31) Papillary thyroid cancer (31)

^1^The reference sequence of the *APC *gene [GenBank:NM_000038] was used. Nucleotide +1 is the A of the ATG-translation initiation codon.

^2^The exon that contained the ATG-translation initiation codon was regarded as the first exon.

^3^When a germline *APC *mutation had been reported previously, the paper reporting it is cited.

^4^Age at diagnosis is shown.

^5^The number of colorectal polyps refers to the cumulative number.

^6^Age at last observation is shown.

^7^NA: not available.

### Detection of abnormal splicing caused by the novel *APC *mutation c.1958G > C

**Figure 2:**
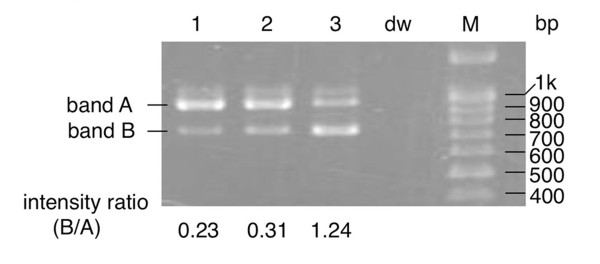
**RT-PCR analysis for evaluation of the effect of the c.1958G > C mutation on splicing**. RT-PCR with a forward primer for the sequence on exon 13 and a reverse primer for the sequence on exon 15 and subsequent agarose gel electrophoresis were performed on blood samples derived from two control subjects with the c.1958G/G genotype (lanes 1 and 2) and a patient with the c.1958G/C genotype (lane 3). The two main bands identified are indicated as band A (upper) and band B (lower). The intensity ratio (intensity of band B/intensity of band A) calculated with ImageJ software is shown under each lane of the panel. dw: distilled water. M: 100-bp ladder.

The novel mutation c.1958G > C detected in Patient 4 was localized in the last nucleotide of exon 14, and three germline *APC *mutations, i.e., c.1956C > T, c.1957A > C, and c.1957A > G very close to c.1958G > C, have previously been reported to induce abnormal splicing of exon 14 [22]. Moreover, a severe reduction in splicing efficiency was predicted for the c.1958G > C mutation by the BDGP splice prediction program, and disruption of the binding site for one of the SR proteins, SC35, by the mutation was predicted by the ESEfinder program for splicing enhancer's motif prediction (Table 2). These predictions prompted us to examine the effect of the c.1958G > C mutation on splicing by mRNA transcript analysis. An RNA sample from the patient with the c.1958G/C genotype and two RNA samples from two control subjects with the c.1958G/G genotype were prepared and used for RT-PCR analysis with a forward primer for the sequence on exon 13 and a reverse primer for the sequence on exon 15. Two main bands were detected in all samples (Figure 2), and direct sequencing of the two bands revealed that the upper band (band A) represented the wild-type fragment and the lower band (band B) represented the whole exon 14-skipped product (data not shown), findings that are consistent with those reported in a previous paper [22]. Calculation of the splicing rate of the mutant-type transcript by dividing the intensity of band B by the intensity of band A with the ImageJ program showed that the splicing rate in samples with the G/C genotype was higher than in samples with the G/G genotype (1.24 vs 0.23 and 0.31) (Figure 2), suggesting that the c.1958G > C mutation caused abnormal splicing.

**Table 2:** Prediction of the effects of the c.1958G > C mutation on splicing

Allele	**Splice prediction**^**1**^	**ESE score for SC35**^**2**^
Wild type (G)	0.91	4.595
Mutant type (C)	< 0.01	-^3^

^1^Predicted by the Berkeley Drosophila Genome Project (BDGP) splice prediction program.

^2^Exonic splicing enhancer (ESE) score as predicted by ESEfinder. Its threshold score for SC35 protein is 2.383.

^3^Below the threshold score.

### Confirmation of the c.454_457insAGAA mutation and the c.3927_3931delAAAGA mutation by MLPA analysis

**Figure 3:**
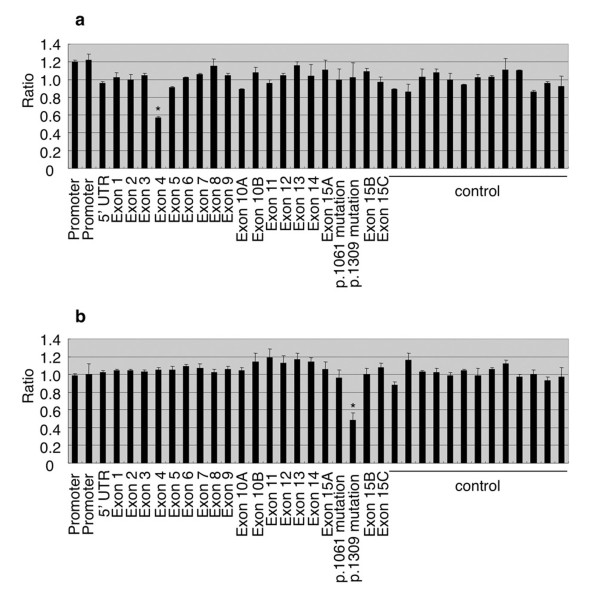
**MLPA analysis for evaluation of the blood DNA of (A)FAP patients**. The results of the MLPA analysis for Patient 2 with a c.454_457insAGAA (p.Glu152ArgfsX17) mutation (a) and Patient 8 with a c.3927_3931delAAAGA (p.Glu1309AspfsX4) mutation (b). Names of MLPA probes are shown below the panels. A decreased signal is indicated by an asterisk. Results are shown as means + standard deviation.

To better evaluate the state of the genomic DNA of (A)FAP patients, MLPA analysis, which is useful for detecting large deletions and duplications, was also performed on all 8 samples. No increased signals were detected, but decreased signals were detected in two patients. One decreased signal was detected in *APC *exon 4 of the DNA derived from Patient 2 (Figure 3a). However, no large deletions were detected in mRNA transcripts from Patient 2 by RT-PCR analysis with a forward primer for the sequence of exon 2 and a reverse primer for the sequence of exon 6 (data not shown). Examination of the sequence of the DNA probe for *APC *exon 4 revealed that the probe for exon 4 overlapped with the c.454_457insAGAA mutation (p.Glu152ArgfsX17) in Patient 2. Thus, it is likely that the overlapping caused the disruption of the MLPA reactions in the DNA. The other decreased signal was detected with the p.1309 mutation-specific probe in Patient 8 (Figure 3b). These results were consistent with the results of the sequencing analysis (p.Glu1309AspfsX4 mutation) in the patient. The results of the MLPA analysis in conjunction with the sequencing analysis indicated that base substitutions or microdeletions, not large deletions or duplications, in the *APC *gene locus caused the production of truncated APC proteins in our (A)FAP patients.

### Characterization of clinical phenotypes of patients with different germline *APC *mutations

The clinical phenotypes of all 8 patients are summarized in Table 1. All 3 patients (Patients 1-3) with a mutation in *APC *exon 4 had been diagnosed with AFAP because of their attenuated colorectal phenotypes. From 40 to 100 colorectal polyps were detected in Patient 1. Patient 2 was found to have a relatively small number of colorectal polyps at 29 years of age, and clinical follow up was performed instead of surgical treatment. At 51 years of age, however, an early-stage CRC and approximately 100 colorectal adenomatous polyps were detected, and total colectomy was performed. A sister of Patient 2 was also diagnosed with AFAP and found to have the same *APC *mutation, but none of the 6 unaffected members of this family was found to have the family-specific mutation. Colon fiberscopy was performed for the first time in Patient 3 when he was 69 years old because of rectal bleeding, and more than 100 colorectal adenomatous polyps and multiple advanced-stage CRCs were observed. Total colectomy was performed, but the patient died of liver and lung metastases 7 years later. By contrast, more than 300 colorectal adenomatous polyps were found in each of the other 5 patients, who were diagnosed with classical FAP. The mother of Patient 4 and the daughter of Patient 5 were found to have their family-specific *APC *mutation, consistent with their FAP phenotype. Among the extracolonic manifestations, gastroduodenal polyps, which are also common in FAP patients, were observed in all of the patients with classical and attenuated FAP in this study. Most of the polyps in the stomach were histologically diagnosed as fundic gland polyps. However, multiple adenomas were observed in the stomach and duodenum of Patients 1, 3, 4, 5, and 7. Desmoid tumors are common in FAP patients and were observed in Patients 5 and 7. Malignant neoplasms in the form of gastric carcinoma, multiple myeloma, duodenal carcinoma, small intestinal carcinoma, and thyroid carcinoma developed in Patient 1, Patient 3, Patient 4, Patient 5, and Patient 8, respectively. No extracolonic manifestations were detected in the other (A)FAP affected members of any of the families, or such information was unavailable.

## Discussion

Sequencing analysis, RT-PCR analysis, and MLPA analysis of the *APC *genes of 8 Japanese (A)FAP patients from 8 unrelated families revealed a nonsense mutation, a frameshift mutation, or an exonic mutation leading to abnormal splicing, all of which resulted in the production of a truncated APC protein, in every patient. No large deletions or duplications in the *APC *locus were detected in any of the patients. Five of the 9 germline *APC *mutations detected in this study had never been reported before, meaning that they are novel mutations. Since two mutations, c.446A > T (p.Asp149Val) and c.448A > T (p.Lys150X), are on the same allele and located close to each other, it is possible that these two mutations are in complete linkage disequilibrium but are not two independent mutations. Consistent with the previous finding that mutations in the 5' and 3' ends and in exon 9 of the *APC *gene are associated with having the attenuated type of FAP [6-10], the mutations in exon 4 were found in the 3 AFAP patients in this study, and the mutations in exons 14, and 15 were found in the 5 classical FAP patients in this study.

Knowledge of genotype-phenotype correlations in (A)FAP has been accumulating, and it is useful in the clinical management of (A)FAP families, but the relationships between the locations of the *APC *mutations and extracolonic manifestations are still not fully understood. Furthermore, as the number of patients diagnosed with (A)FAP has increased, a broad range of variable extracolonic manifestations has gradually come to be recognized in (A)FAP patients. Based on the results of *APC *mutations and extracolonic manifestations observed in our study, we focused our attention on the following three issues. First, fundic gland polyps, not gastric adenoma, have been reported to be the main gastric lesions in AFAP patients [1,8]. Interestingly, multiple adenomas in the stomach, in addition to the commonly detected fundic gland polyps, as well as in the duodenum were observed in 2 of our 3 AFAP patients with germline mutations in *APC *exon 4, and a gastric adenocarcinoma was also found in one of them at 34 years of age. As far as we know [7,8], this is the first report of such an early-onset gastric cancer in AFAP patients. Gastric adenocarcinomas have been reported in (A)FAP patients [23-26], but the mechanism of involvement of the *APC *mutation in the gastric carcinogenesis remains largely unknown. There have been reports of gastric adenocarcinomas in (A)FAP patients having arisen from a fundic gland polyp or adenoma, and the reports suggested the existence of an adenoma-adenocarcinoma sequence in their carcinogenesis [23-25]. However, there have been other reports claiming to have found no relation between a gastric adenocarcinoma in an AFAP patient and existing fundic gland polyps or adenomas [26]. Analysis of the *APC *inactivation status in the adenomas and adenocarcinomas detected in our patients may help to better understand the mechanism of the involvement of *APC *mutations in tumorigenesis. In addition to genetic factors, various environmental factors have been reported to affect the risk of gastric cancer [27-29], environmental factors and genes that modify the development of gastric lesions may be involved in the clinical phenotype. The second issue is that it has previously been reported that there is a close association between the presence of a germline *APC *mutation between codons 1403 and 1578 in FAP patients and the occurrence of desmoid tumors [1,3], however, both of the FAP patients with a desmoid tumor in our series had a germline *APC *mutation outside the region, i.e., in codon 664 and codon 1249. Some other recent papers have also reported the occurrence of desmoid tumors in FAP patients with *APC *mutations outside the region between codons 1403 and 1578 [30,31]. Thus, the results of this study and others together with the findings described in previous reviews [1,3] have suggested that although FAP-associated desmoids predominantly occur in patients carrying *APC *mutations between codons 1403 and 1578, some of them occur outside the region. The third issue that we focused our attention on is that a multiple myeloma was detected in one of our 3 AFAP patients with a germline exon 4 mutation. This is the first report of complication of AFAP by multiple myeloma. However, evidence of *APC *involvement, such as a second somatic mutation of *APC *in multiple myeloma, needs to be found in order to rule out the possibility that the occurrence of the multiple myeloma was a coincidental unrelated event. We think that although the number of cases analyzed was relatively small, the above three findings will contribute to establishing relationships between germline *APC *abnormalities and clinical phenotypes in (A)FAP patients and to better characterizing the differences between APC-related polyposis and MutYH-associated polyposis in the future. However, since many examples of deviations from observed *APC *genotype-FAP phenotype correlations and highly variable phenotypic traits have been reported [16,32-34], it has been pointed out that the family history is important in (A)FAP genotype-phenotype analyses and the factors other than the *APC *genotype may be involved in producing the (A)FAP phenotype.

Although a nearly 100% risk of CRC has been reported in patients with classical FAP, the lifetime risk of CRC in AFAP patients is unclear. At the time this study was performed CRC had been diagnosed in Patient 2 at 51 years of age and Patient 3 at 69 years of age, but not in Patient 1, who was 38 years old. Lifetime risk of CRC is an important factor in the clinical management and genetic counselling of AFAP patients, especially because it affects the decision as to whether to proceed with prophylactic colectomy. The results of this study should contribute to determining the lifetime risk of CRC in AFAP patients in the future.

The results of the RT-PCR analysis in this study showed that a G to C transversion at c.1958, which corresponds to the last nucleotide of exon 14 of the *APC *gene, causes abnormal splicing. Since APC mRNA transcript analyses in previous studies have demonstrated that exonic single-base substitutions of c.423G > T, c.834G > C, c.1869G > T, c.1918C > G, c.1956C > T, c.1957A > C, and c.1957A > G affect splicing [22,35-37], c.1958G > C is the 8th *APC *exonic mutation that has been demonstrated to result in abnormal splicing. Interestingly, most (6 out of 8) of the exonic mutations associated with abnormal splicing have been located in exon 14, but the reason for this clustering is unclear.

## Conclusions

In the present study, 9 germline *APC *mutations, 5 of which were novel, were identified in 8 Japanese (A)FAP patients. The following three findings regarding the relation between the location of the germline *APC *mutations and extracolonic manifestations were also obtained in this study: 1) severe gastric lesions occurred in AFAP patients with an exon 4 mutation, 2) desmoid tumors developed in FAP patients with germline *APC *mutations outside the region between codons 1403 and 1578, 3) a multiple myeloma developed in an AFAP patient with an exon 4 mutation. These findings should contribute to increasing our knowledge of the associations between *APC *genotypes and (A)FAP phenotypes, which are informative for proper clinical management and genetic counselling of (A)FAP patients and their families.

## Competing interests

The authors declare that they have no competing interests.

## Authors' contributions

HT carried out the mutation detection and molecular genetic analyses, collected up the clinical data, participated in the design of the study, and drafted the manuscript. KS participated in the design of the study and drafted the manuscript. HY extracted the total RNAs from blood samples. MM, SO, YT, KO, TT, HM and TN identified relevant patients for genetic testing and were responsible for clinicopathological data. HS and MM conceived of the study, participated in the design of the study, and drafted the manuscript. All authors read and approved the final manuscript.
